# Growth and life history variability of the grey reef shark (*Carcharhinus amblyrhynchos*) across its range

**DOI:** 10.1371/journal.pone.0172370

**Published:** 2017-02-16

**Authors:** Darcy Bradley, Eric Conklin, Yannis P. Papastamatiou, Douglas J. McCauley, Kydd Pollock, Bruce E. Kendall, Steven D. Gaines, Jennifer E. Caselle

**Affiliations:** 1 Bren School of Environmental Science and Management, University of California Santa Barbara, Santa Barbara, California, United States of America; 2 The Nature Conservancy, Honolulu, Hawaii, United States of America; 3 Department of Biological Sciences, Florida International University, North Miami, Florida, United States of America; 4 Department of Ecology, Evolution, and Marine Biology, University of California Santa Barbara, Santa Barbara, California, United States of America; 5 Marine Science Institute, University of California Santa Barbara, Santa Barbara, California, United States of America; Department of Agriculture and Water Resources, AUSTRALIA

## Abstract

For broadly distributed, often overexploited species such as elasmobranchs (sharks and rays), conservation management would benefit from understanding how life history traits change in response to local environmental and ecological factors. However, fishing obfuscates this objective by causing complex and often mixed effects on the life histories of target species. Disentangling the many drivers of life history variability requires knowledge of elasmobranch populations in the absence of fishing, which is rarely available. Here, we describe the growth, maximum size, sex ratios, size at maturity, and offer a direct estimate of survival of an unfished population of grey reef sharks (*Carcharhinus amblyrhynchos*) using data from an eight year tag-recapture study. We then synthesized published information on the life history of *C*. *amblyrhynchos* from across its geographic range, and for the first time, we attempted to disentangle the contribution of fishing from geographic variation in an elasmobranch species. For Palmyra’s unfished *C*. *amblyrhynchos* population, the von Bertalanffy growth function (VBGF) growth coefficient *k* was 0.05 and asymptotic length *L*_∞_ was 163.3 cm total length (TL). Maximum size was 175.5 cm TL from a female shark, length at maturity was estimated at 116.7–123.2 cm TL for male sharks, maximum lifespan estimated from VBGF parameters was 18.1 years for both sexes combined, and annual survival was 0.74 year^-1^. Consistent with findings from studies on other elasmobranch species, we found significant intraspecific variability in reported life history traits of *C*. *amblyrhynchos*. However, contrary to what others have reported, we did not find consistent patterns in life history variability as a function of biogeography or fishing. Ultimately, the substantial, but not yet predictable variability in life history traits observed for *C*. *amblyrhynchos* across its geographic range suggests that regional management may be necessary to set sustainable harvest targets and to recover this and other shark species globally.

## Introduction

Intraspecific variation has been observed in many life history traits (e.g. growth, maximum size, size at maturity, fecundity) for a number of elasmobranchs (sharks and rays) [1–13], but the drivers of this variability are often unclear. Many elasmobranchs have broad, even circumglobal distributions, and therefore experience regional differences in environmental conditions, ecological factors, and anthropogenic stressors, all of which directly affect life history traits [14]. However, while body size and size at maturity tend to increase with latitude for bony fishes, the relationship between life history variability and latitudinal variation is less clear in sharks [3,6,12,13]. At the same time, many shark populations have been severely depleted by fishing and are considered overfished [15–20], making it difficult to disentangle changes to population parameters due to geographic variability, ecological variability, and anthropogenic impacts.

For broadly distributed species such as elasmobranchs, conservation management would benefit from understanding how life history traits change in response to local environmental and ecological contexts. Unfortunately, such information is generally lacking. In fact, nearly half of the chondrichthyan species (sharks, rays, and chimaeras) are considered ‘Data Deficient’, that is, lacking requisite information to assess their status by Red List Categories and Criteria of the International Union for the Conservation of Nature [19]. Discerning context-specific effects on life history traits is further complicated by the fact that fishing causes complex and often mixed effects on the life histories of target species. For example, fishing can both increase fecundity via density dependent effects [21] or depress fecundity via size selective harvest [22]. Fishing can also have complicated effects on mortality rates [23], maximum size [1,2], size and age at maturity [2,24,25], and growth rates [1,2,22]. Understanding how fishing has altered life history traits requires knowledge of populations in the absence of fishing, which is rarely possible.

Like most fishes, sharks grow deterministically and the von Bertalanffy growth function (VBGF; [26]) is often selected as the most appropriate growth model for sharks [27–29]. VBGF parameters are also used as proxies in estimations of life history parameters, including natural mortality, in fisheries stock assessments [30,31]. A biased estimate of growth can therefore bias life history estimates, resulting in inaccurate assessments of stock status (e.g. [32]). This is problematic, because stock assessments depend on accurate life history information to set harvest targets.

Although no area of the world’s ocean is unaffected by human influence [33], Palmyra Atoll in the northern Line Islands is considered a little-disturbed ecological reference site that provides an opportunity to study a coral reef ecosystem without significant human impacts, including extractive fishing pressure. Palmyra is a remote, historically uninhabited, U.S. National Wildlife Refuge in the central Pacific Ocean (5°54’N; 162°05’W) that was established in 2001. Prior to receiving federal protection, Palmyra was privately owned for over 100 years, and only briefly housed a permanent human population when it was occupied by the U.S. Navy during World War II. Although Palmyra’s lagoons were significantly impacted during Naval occupation, its outer reefs were left nearly undisturbed [34]. Under current management, commercial fishing and extractive recreational fishing are banned within 50 nautical miles of Palmyra. The uniqueness and ecological value of Palmyra’s unfished marine ecosystem has attracted considerable research attention, and researchers have shown that Palmyra’s reefs are home to a significantly higher biomass of sharks than neighboring, inhabited islands, the nearest of which is 230 km away [35,36]. Grey reef sharks (*Carcharhinus amblyrhynchos*) are the most abundant shark species on Palmyra in terms of biomass [37], and as such are the flagship species of the refuge. Ongoing work is showing that Palmyra supports a large and temporally stable population of these predators (Bradley, unpublished data).

*Carcharhinus amblyrhynchos* is a reef-associated shark species, broadly distributed throughout the Indian, and western and central Pacific Oceans [38,39]. While adult female grey reef sharks generally reach larger sizes than males, there is a high level of variability in reported maximum total length (TL). In 13 published studies with a minimum sample size of 25 sharks, adult *C*. *amblyrhynchos* maximum TL varies by region from 152.5 cm in the Line Islands (male; [40]) to 200 cm in the Marshall Islands (sex not reported; [41]), with a maximum reported size for the species of 255 cm TL [42]. Regional variation is also evident in life history analyses conducted on *C*. *amblyrhynchos* in the Northwestern Hawaiian Islands (NWHI) [43], the northern and central Great Barrier Reef (GBR) [44], and Papua New Guinea (PNG) [45], which encompass a gradient of environmental, ecological, and anthropogenic impacts. Including Palmyra, these regions span 2–28° latitude and have different ecological characteristics. *C*. *amblyrhynchos* is the most abundant reef-associated predator in terms of biomass at Palmyra [37], the GBR [44,46], and PNG (based on commercial fisheries landings data [47]), but not in the NWHI [48]. Competition and predation are therefore expected to exert different selection pressures on *C*. *amblyrhynchos* in each location. However, fishing pressure also varies regionally, with recently active commercial shark fisheries that target *C*. *amblyrhynchos* in PNG [45] and the GBR [49,50], and no shark fisheries in NWHI [51] and Palmyra. The key challenge is disentangling the contributions of fishing from geographical variation in the biological and physical characteristics of these ecosystems. One barrier to this effort is the absence of data from multiple sites with no history of fishing.

Here, we describe the growth, maximum size, sex ratios, length at maturity, and offer a direct estimate of natural mortality and survival of an unfished population of *C*. *amblyrhynchos* using data from an eight year tag-recapture study. We then synthesize published information on the life history of *C*. *amblyrhynchos* from across its geographic range, and for the first time, we attempt to disentangle the contribution of fishing from geographic variation in an elasmobranch species.

## Methods

### Ethics statement

This project has been certified by the Institutional Animal Care and Use Committee (IACUC), University of California, Santa Barbara, Protocol no. 856 (date of IACUC approval: 5/31/2012). Sharks were captured at Palmyra Atoll, which has been a U.S. National Wildlife Refuge since 2001 and part of the Pacific Remote Islands Marine National Monument since 2009, under U.S. Fish and Wildlife Service special use permits (Permit numbers #12533–14011, #12533–13011, #12533–12011, #12533–11007, #12533–10011, #12533–09010, #12533–08011, and #12533–07006).

### Data collection

From October 2006 to October 2014, we captured and tagged *C*. *amblyrhynchos* in the forereef, backreef, lagoon, and channel habitats on Palmyra. Sharks were caught using hand lines baited with yellowfin tuna (*Thunnus albacares*), wahoo (*Acanthocybium solandri*), and/or mackerel (*Scomber scombras* and *Decapterus macrosoma*) on barbless circle hooks. Once captured, individuals were restrained at the side of the boat using a tail rope. Up to three length measurements were recorded to the nearest 0.5 cm for each shark: precaudal length (PCL), fork length (FL), and TL (in accordance with the FAO shark measurement protocol [52]). Measurements were taken in the same way throughout the study by running a measuring tape along the dorsal side of each animal. Sex was determined by the presence/absence of claspers, which were measured, and clasper state was assessed to determine maturity for all male individuals (calcified, partially calcified, not calcified [53]). Maturity estimates for female sharks were not made from direct field observations. Uniquely numbered tags were then affixed to individual sharks. Early in the study, rototags were applied with an applicator through a hole punched in the leading edge of the first dorsal fin. Starting in 2010, minimally invasive Hallprint^™^ dart tags with stainless steel heads were applied using stainless steel tag applicators, with the tag head implanted in the epaxial muscle near the base of the first dorsal fin. Handling time was <4 minutes on average for an individual shark and we found no evidence of tag loss during the study period, (for details see [54]).

### Statistical analyses

We used t-tests to compare the average size of captured female and male *C*. *amblyrhynchos*, and chi-squared tests to assess whether the observed sex ratio was significantly different from 1:1 (*α* = 0.05). Length at maturity *TL*_*m*_ for male sharks was estimated by logistic regression using the *glm* function with a binomial error distribution in R [55], solving for the total length at which 50% of males had calcified claspers (odds of calcified clasper = 1; odds of not calcified or partially calcified clasper = 0). We obtained 95% confidence intervals for regression coefficient estimates by taking 10,000 bootstrap samples. For comparison, length at maturity was also estimated for male sharks using the published elasmobranch-wide linear relationship between *TL*_*m*_ and maximum length *TL*_*max*_ of a captured individual (*TL*_*max*_ = 175.5 cm at Palmyra) [56] (S1 Appendix). In addition, length-to-length conversion formulae (i.e. TL-PCL, TL-FL, FL-PCL, etc.) were estimated using linear regression analyses. Including gender in the slopes and intercepts of length-to-length conversion models decreased model fit (assessed using Akaike’s information criterion [57] (AIC) with improved model fit indicated by a ΔAIC value >3 with the addition of model parameters [58]), and so we report only models with sexes combined.

Using our capture-recapture data, we fit the Francis [59] formulation of the VBGF for female sharks and with both sexes combined (due to our limited sample of recaptured male sharks). Previous studies have found nearly identical growth curves in male and female requiem sharks [60,61] including *C*. *amblyrhynchos* [44,45]. For sharks recaptured on multiple occasions, the initial length and final length measurements and their corresponding dates were included in the growth analyses. The Francis [59] model includes parameters *g*_*α*_ and *g*_*β*_, which are the mean annual growth increments of a species at the arbitrary reference lengths *α* and *β*, which can be used to estimate the conventional VBGF parameters *L*_∞_ and *k* (S1 Appendix). Francis [59] model *L*_∞_ and *k* values estimated from tagging data have the same biological meaning as VBGF parameters derived from age-length data [59]: *L*_∞_ (cm) is the asymptotic mean length at age (i.e. the average length of an “old” shark), and *k* (year^-1^) is a growth coefficient that describes the rate at which growth approaches *L*_∞_. The Francis [59] model is also more flexible than other VBGF methods for tagging data in that it allows the inclusion of additional parameters that can affect model fit, including the coefficient of variation of growth variability (*v*), mean (*m*) and standard deviation (*s*) of measurement error, outlier contamination (*p*), and seasonal variation (*u*, *w*). Several combinations of additional parameters were considered using a stepwise fitting procedure, but we only present the best fitting parameterizations. Only recaptured individuals with minimum time at large of 150 days were included in the growth analysis. Previous studies of *C*. *amblyrhynchos* used TL [44,45,62] and PCL [43] to generate growth estimates; PCL was not measured at every sampling occasion in this study and so TL was used instead. However, *FL*_∞_ and *PCL*_∞_ were estimated from *TL*_∞_ using the length-to-length conversions described above. All Francis [59] model parameters were estimated using the R package *fishmethods* [63]. Maximum lifespan *T*_*max*_ for sexes combined was then estimated as the time required to attain >99% of *TL*_∞_ as *T*_*max*_ = 5∙Ln(2)∙*k*^-1^ [64].

Model selection was performed on our different parameterizations of the Francis [59] model using likelihood ratio tests (improved model fit indicated by a likelihood value >1.92 for one additional parameter and >3.0 for two additional parameters [59]) and by comparing AIC values (as above). We also visually assessed model fit by plotting model residuals (observed-expected growth) against length at release and predicted growth; residual deviation was expected to decrease as length at release increases (*L1*), because the likelihood function assumes an allometric relationship between individual growth variation and mean growth, and the latter declines with length [65]. Residual deviation was also expected to positively correlate with predicted growth based on the assumed relationship for individual growth variability (S1 Appendix). We used *C*. *amblyrhynchos* reference lengths *α* (100 cm TL) and *β* (130 cm TL) to estimate *k* and *TL*_∞_ (S1 Appendix). Reference lengths should lie within range of length at capture data, and should have different associated growth rates, but their values are otherwise arbitrary [59]. To ensure that the model was performing well, ranges of *α* (95–105 cm) and *β* (125–145 cm) values were examined, but parameter estimates were insensitive to values within these ranges. These *k* and *TL*_∞_ parameters were then used to generate a growth curve for unfished grey reef sharks at Palmyra Atoll (sexes combined). As the Francis [59] model for tagging data does not provide an estimate of size at birth *L*_0_ (S1 Appendix), we assumed that *L*_0_ = 60 cm for Palmyra sharks [39,62] to generate our growth curve and growth curves for existing studies using the VBGF *L*_*t*_ = *L*_∞_ + (*L*_∞_ − *L*_0_)*e*^−*kt*^ [26]. Capture-recapture data used to estimate our growth model is presented in S1 Table.

We note that previous authors have suggested a multimodel approach to estimate growth for sharks [12,29], particularly for tag-recapture samples [12]. For this analysis, other growth methods were considered (e.g. Gulland and Holt [66], Fabens [64], Fabens with a fixed asymptote [67]), but none were deemed as appropriate for our data as the Francis [59] method. The Fabens [64] method is known to produce biased estimates of *k* and *L*_∞_ when individual growth is variable [68–71] or measurement error is high [72], indicating that the Fabens [64] model would likely overestimate length at time for our data [68]. Previous studies have found that the Gulland and Holt [66] method produces growth estimates that are sometimes similar to, but often less biologically realistic than, the Francis [59] method [61,68,73,74]. The Francis [59] method is certainly not free from bias, but it is generally the least biased of the commonly used growth models for tag-recapture data [12], and it has been found to be a better fit than other methods for other requiem shark species [61,74].

Francis [69] suggested that growth at length comparisons between length-increment and age-length data studies is more appropriate than direct parameter comparisons across studies. Therefore, we calculated growth at length TL_130_ for each existing *C*. *amblyrhynchos* study using the VBGF reported above [26], where *t* = 1. Existing *C*. *amblyrhynchos* age and growth studies were consistent in estimating individual ages by counting translucent and opaque bands in the corpus calcareum, and all assumed annual growth band deposition [43–45]. In two of the three existing studies, individuals were independently aged 2–3 times and either tests for systematic bias or independent external confirmation of age estimates were undertaken to ensure precision [44,45]. Although band count variability between readers and labs can bias elasmobranch age and growth studies [75,76], particularly in terms of age underestimation [77], discrepancies in Carcharhiniformes tend to be minimal (as compared to Lamniformes, for example) [78]. Smart et al. [45] determined that the average percent error for *C*. *amblyrhynchos* growth bands between readers was 9.46%, which is expected for long-lived species [76], and not large enough to affect our comparisons. Previous authors have also shown consistency between Francis [59] growth at length from tagging data and growth at length estimated from age-length data for sharks [79].

Finally, we directly estimated survival *ϕ* (and therefore mortality) and derived estimates of total instantaneous mortality *Z* for *C*. *amblyrhynchos* at Palmyra Atoll, with the expectation that Z *= M* given the absence of fishing. Tag-recapture data was used to formulate a restricted dynamic occupancy version of the Jolly-Seber (JS) model [80] to estimate probability of annual survival (S1 Appendix). We used a Bayesian analysis and specified a uniform prior U(0,1) on our survival parameter. Our model was estimated using data augmentation with Markov chain Monte Carlo (MCMC) sampling in the R package *rjags* [81]. We ran 3 chains for 25,000 iterations and discarded the first 5,000 runs as burn-in. We examined traceplots and the Gelman-Rubin $\hat{r}$ statistic (values <1.1 indicate convergence) to assess model performance. Total mortality was then estimated using the Hoenig [82] equation (*Z* as a function of *T*_*max*_; S1 Appendix) parameterized separately for teleost fishes and for cetaceans. The parameterization for cetaceans is often used for sharks because cetaceans have demographic characteristics more similar to sharks than teleosts [83]. All statistical analyses were performed in R [55].

## Results

**Fig 1:**
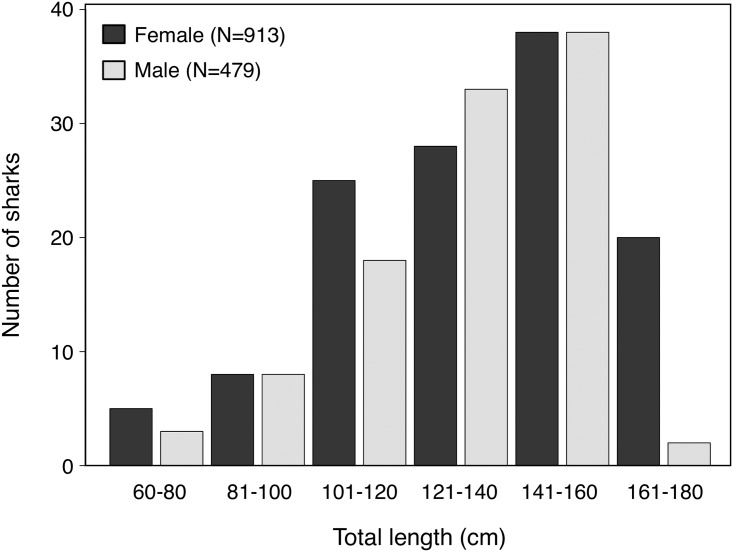
Total length frequency of female and male *C*. *amblyrhynchos* captured by research fishing in Palmyra 2006–2014.

**Fig 2:**
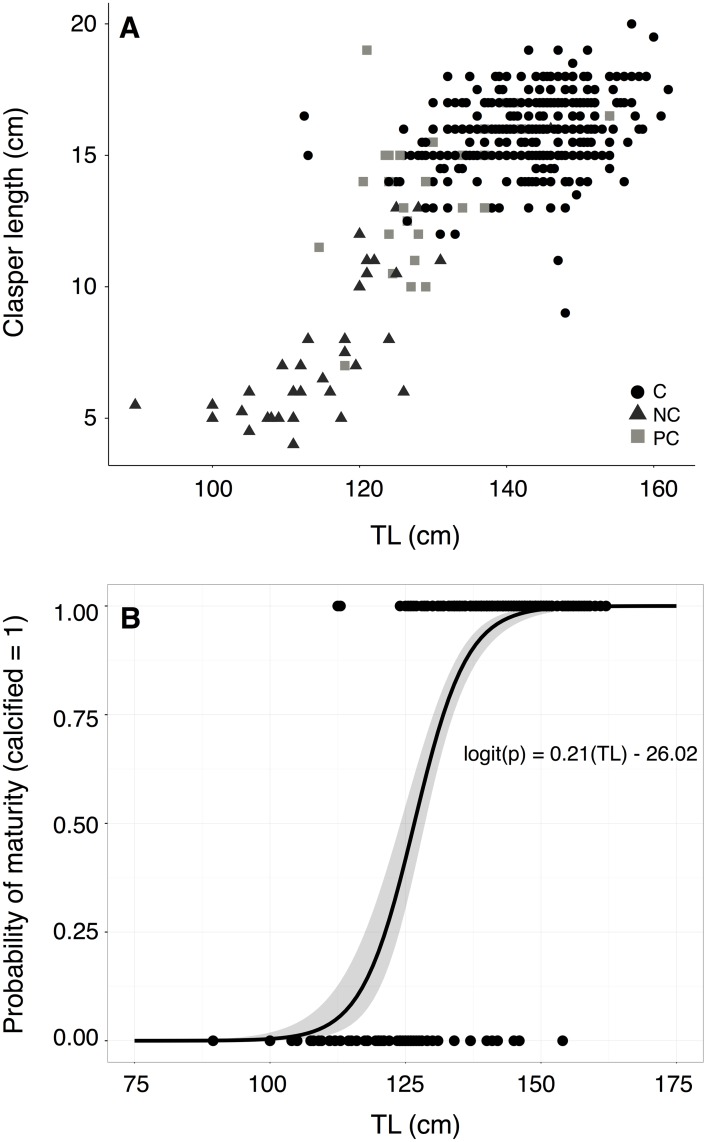
Grey reef male maturity. (A) Clasper size (cm) as a function of TL; NC = not calcified (N = 35); PC = partially calcified (N = 33); C = calcified (N = 361). (B) Only calcified (C = 1) and not calcified (NC = 0) individuals were included in the logistic regression.

We captured 1399 individual *C*. *amblyrhynchos* by research fishing on Palmyra between 2006–2014; of these, 1356 individuals were tagged with either a dart and/or roto tag. The sample was slightly female biased (male:female ratio = 0.52, *χ*^2^ = 135.8, p<0.001), and female sharks were significantly larger than male sharks (Fig 1). The average captured female was 146.0 cm TL (SD = 16.6 cm) while the average captured male was 138.7 cm TL (SD = 14.2 cm) (*t*_1109.8_ = 8.6; *p*<0.001). The largest captured shark was a 175.5 cm TL female, and the smallest captured individuals were four 66 cm TL females. Length-to-length relationships had conversion estimates with *R*^*2*^ > 0.90 (Table 1). The average captured immature male shark was 123.0 ± 1.5 cm (mean ± SE), and the average captured mature male shark was 143.5 ± 0.4 cm (Fig 2A). Male *C*. *amblyrhynchos* had a 50% chance of reaching maturity at 123.2 cm TL (logistic regression, p < 0.001, 95% CI 123.0–123.7 cm TL) (Fig 2B). Length at maturity *TL*_*m*_ estimated as a function of *TL*_*max*_ [56] was 116.7 cm for male sharks.

**Table 1:** Length-to-length relationships for *Carcharhinus amblyrhynchos* (sexes combined) captured by research fishing in Palmyra 2006–2014.

x	y	b_0_	b_1_	r^2^	df	p
TL	FL	-1.12(0.71)	0.85(0.00)	0.96	1390	<0.001
TL	PCL	-6.37(0.96)	0.79(0.01)	0.93	1115	<0.001
FL	TL	7.67(0.79)	1.13(0.01)	0.96	1390	<0.001
FL	PCL	-1.74(0.88)	0.91(0.01)	0.93	1114	<0.001
PCL	TL	17.88(1.06)	1.17(0.01)	0.93	1115	<0.001
PCL	FL	9.78(0.89)	1.03(0.01)	0.93	1114	<0.001

Linear regression coefficients for the model *y*_*i*_ *= b*_*0*_ *+ b*_*1*_*x*_*i*_.

Numbers in parentheses are standard errors.

PCL, pre-caudal length (cm); FL, fork length (cm); TL, total length (cm).

**Fig 3:**
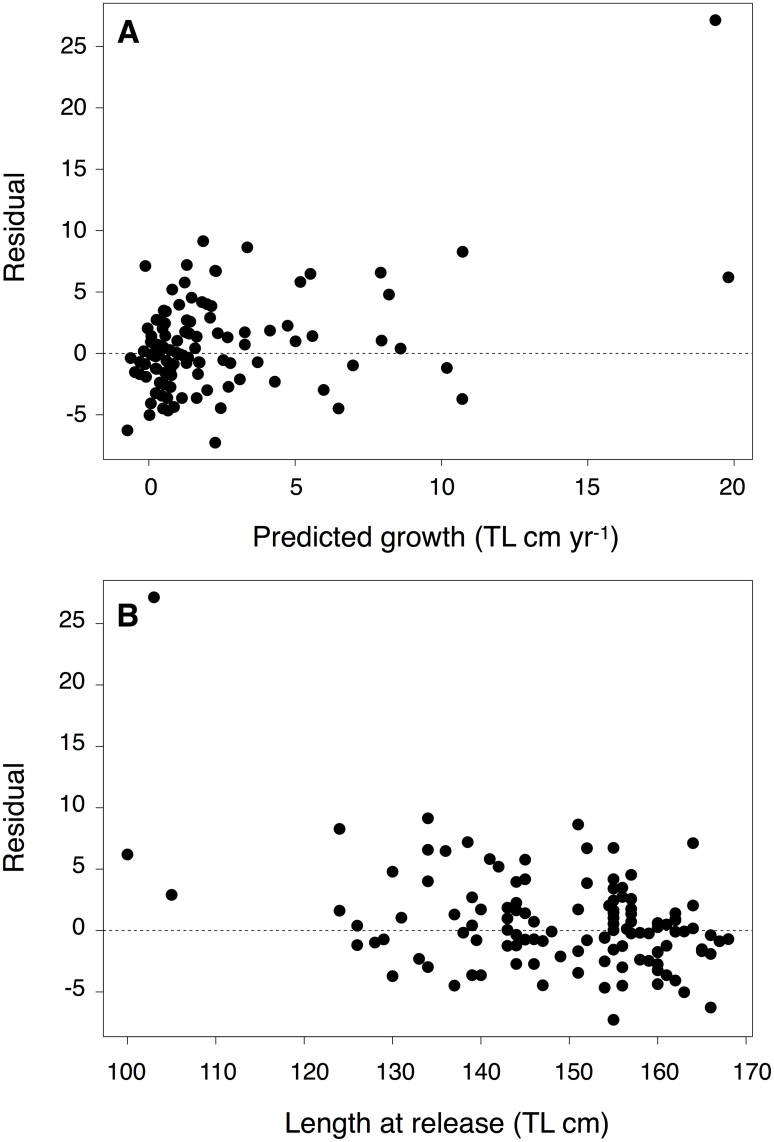
Residual plots to assess Francis [59] model fit. Francis growth model residuals (observed-expected growth) plotted against (A) predicted growth (TL (cm ∙ yr^-1^) for *C*. *amblyrhynchos*), and (B) length at release (cm); residual deviation was expected to decrease as length at release increases (*L1*), because the likelihood function assumes an allometric relationship between individual growth variation and mean growth, and the latter declines with length.

**Fig 4:**
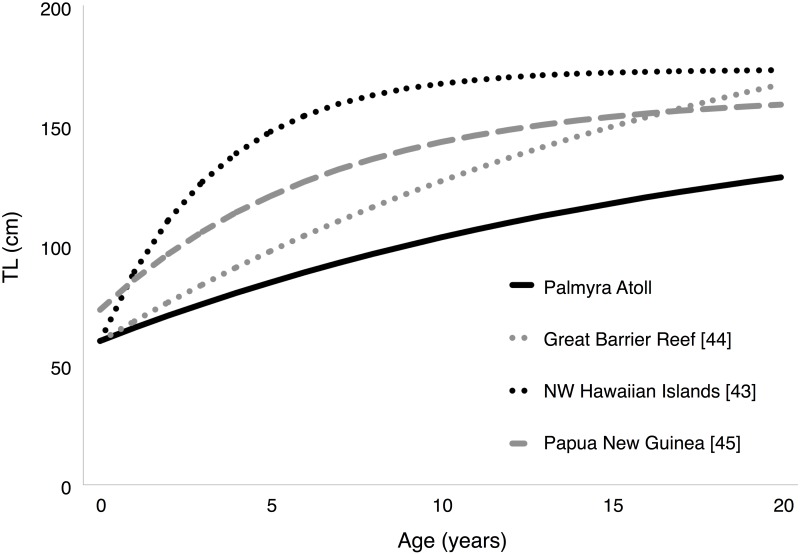
Von Bertalanffy growth curves for Palmyra *C*. *amblyrhynchos* compared to other regions (NWHI, GBR, PNG); both sexes combined. Size at birth based on what is reported in each corresponding reference.

Of the 1356 captured and tagged *C*. *amblyrhynchos*, 118 individuals were recaptured and released at Palmyra a minimum of 150 days after initial capture (females = 100; males = 18). Median time at large was 2.0 years and the longest time between release and recapture was 7.2 years (S1 Table). The maximum total growth recorded for a shark was 46.5 cm from a female captured at 103 cm TL and recaptured 6.8 years later at 149.5 cm TL (S1 Table). The assumptions of the Francis [59] model were confirmed by plotting residuals against length at release and predicted growth. As expected, residual deviance increased over predicted growth and decreased over length at release, reinforcing the expectation of the VBGF that growth variability should decline at increasing length (Fig 3). The female-only and combined-sexes Francis [59] growth model produced consistent results, indicating that including male sharks did not bias model estimates or increase standard errors. The growth model parameterization with the best fit to the VBGF for our capture-recapture data included *g*_*α*_ (*g*_100_), *g*_*β*_ (*g*_130_), standard deviation of measurement error, and growth variability. The inclusion of parameters for mean of measurement error, seasonal variability, and outliers did not significantly improve model fit (Table 2). Growth at length was estimated at 3.33 cm/year (*g*_100_) and 1.75 cm/year (*g*_130_) for our best model (Table 2). The growth coefficient *k* was 0.054 and *TL*_∞_ was 163.3 cm (Table 2). A size at birth of 60 cm, reported in [39,62], was used to fit a von Bertalanffy [26] growth curve to the data (Fig 4). Using the growth coefficient *k* estimated from the Francis model with sexes combined, longevity *T*_*max*_ was 18.06 years. Annual survival rate estimated from tag-recapture data was 0.739 year^-1^ (95% CI 0.703–0.775; $\hat{r}$ = 1.04). Total mortality *Z* from the Hoenig [82] equation was 0.232 year^-1^ and 0.205 year^-1^ (for teleost and cetacean parameterizations, respectively).

**Table 2:** Parameter values from the best fit estimated Francis [59] growth models for *Carcharhinus amblyrhynchos* at Palmyra Atoll.

				Parameter estimates ^a^									
Sex	Model	Likelihood	AIC	*g100* (cm/year)	*g130* (cm/year)	*u* (year)	*w* (year)	*v*	*s* (cm)	*m* (cm)	*p*	*TL*_∞_ (cm)	*k*
**Female**	1	-284.2	574.3	3.65(0.57)	1.87(0.27)	0^b^	0^b^	0^b^	4.15(0.38)	0^b^	0^a^	161.41	0.061
	**2**^c^	**-261.2**	**530.3**	**3.55(0.79)**	**1.87(0.38)**	**0**^b^	**0**^b^	**0.63(0.26)**	**2.82(0.26)**	**0**^b^	**0**^a^	**163.21**	**0.058**
	3	-260.2	530.4	3.76(0.73)	1.87(0.37)	0^b^	0^b^	0.57(0.20)	2.90(0.26)	0.76(0.62)	0^a^	159.60	0.065
	4	-260.2	532.4	3.76(0.73)	1.87(0.38)	0^b^	0^b^	0.57(0.21)	2.90(0.29)	0.76(0.64)	0.0(0.17)	159.6	0.065
	5	-260.2	536.3	3.76(0.72)	1.87(0.38)	0.44(0.0)	0.0(0.0)	0.56(0.21)	2.90(0.29)	0.75(0.64)	0.0(0.17)	159.61	0.065
**Both**	1	-334.9	675.8	3.46(0.48)	1.75(0.23)	0^b^	0^b^	0^b^	4.13(0.35)	0^b^	0^a^	160.71	0.059
	**2**^c^	**-310.2**	**628.3**	**3.33(0.75)**	**1.75(0.36)**	**0**^b^	**0**^b^	**0.66(029)**	**2.87(0.25)**	**0**^b^	**0**^a^	**163.3**	**0.054**
	3	-309.1	628.3	3.50(0.02)	1.75(0.02)	0^b^	0^b^	0.59(0.15)	2.95(0.23)	0.65(0.32)	0^a^	160.01	0.060
	4	-309.2	630.3	3.51(0.70)	1.75(0.36)	0^b^	0^b^	0.58(0.23)	2.95(0.30)	0.67(0.54)	0.0(0.16)	159.88	0.060
	5	-309.0	634.0	3.51(0.0)	1.75(0.0)	1.0(0.0)	0.0(0.0)	0.58(0.09)	2.95(0.24)	0.65(0.0)	0.0(0.0)	159.97	0.060

^a^ Francis model parameters: g100 and g130 = mean annual growth increments at reference lengths 100cm and 130 cm; *u* and *w* = seasonal variation; *v* = growth variability; *s* = standard deviation of measurement error; *m* = mean of measurement error; *p* = outlier contamination.

^b^ Parameters held fixed

^c^ Best model

## Discussion

Consistent with findings from studies on other elasmobranch species, we found significant variation in life history traits of *C*. *amblyrhynchos* across its geographic range. However, contrary to what others have reported [1–3,6,13,22,24,25], we did not find consistent patterns in life history variability as a function of geographic variation or fishing. Previous studies have demonstrated that asymptotic length and growth rate have a positive relationship with latitude in some elasmobranch species [6,12]. However, the relationship between these life history traits and latitude was not consistent for *C*. *amblyrhynchos*. Growth at length was indeed highest in the highest latitude location (NWHI, 23.1–28.2° N) [43], but was also relatively large in PNG (2–11° S) [45], a low latitude location (Table 3; Fig 4). In addition, expected geographic trends in *L*_∞_ were not observed, with the highest *TL*_∞_ reported from the GBR, which was not the highest latitude location (Table 3). There was also no latitudinal pattern in body size (maximum total length) for *C*. *amblyrhynchos* from studies across the species’ Indo-west and central Pacific range (Table 4). Female sharks generally reach larger maximum sizes than male *C*. *amblyrhynchos* (Table 4), a pattern that was also observed at Palmyra (Fig 1). However, *C*. *amblyrhynchos* in the largest size classes that have been reported elsewhere (>180cm [39]) were not encountered in our Palmyra sample. We offer the hypothesis that local abiotic factors may exert more selection pressure on the life history of *C*. *amblyrhynchos* than abiotic factors that follow a latitudinal cline. There are substantial differences in habitat availability, habitat complexity, and connectivity in Palmyra, the NWHI, PNG, and the GBR, and the physical structure of coral reefs plays a fundamental role in determining the strength of biological interactions and the allocation of available energy to life history traits [84].

**Table 3:** Parameter values estimated for growth models for *Carcharhinus amblyrhynchos*.

Location	Latitude	*TL*_∞_ (cm)	*FL*_∞_ (cm)	*PCL*_∞_ (cm)	k	*g130* (cm/year)	N	Method	Reference
GBR Australia	14.7–19.3° S	229.2	188.61^a^	173.33^a^	0.05	5.08	89	VBGF [26]	[44]
Papua New Guinea	2.0–12.0° S^b^	163	--^c^	--	0.15	5.33	133	VBGF [26]	[45]
NW Hawaiian Islands	23.1–28.2° N	173.3^d^	--	134	0.29	14.68	62	VBGF [26]	[43]
Palmyra Atoll	5.8–5.9° N	163.3	137.7^e^	122.7^e^	0.05	1.75	118	Francis [59]	This study

^a^ Calculated using length-to-length relationship reported in [44]

^b^ Minimum and maximum values for Bismarck and Solomon Seas; no latitude values reported in [45]

^c^ “--” indicates an unavailable value

^d^ Calculated using length-to-length relationship reported for Hawaiian C. amblyrhynchos in [39]: TL = 4.146 + 1.262PCL

^e^ Calculated using length-to-length relationships reported in this study

**Table 4:** Life history characteristics for *Carcharhinus amblyrhynchos* from across its geographic range (only studies with a minimum of N = 25 sharks were included).

Location	Latitude^a^	Max length (cm) [sex]	Maturity (cm) [sex]	Length at birth (cm)	Litter size	N	Sample	Reference
Australia (central GBR)	18.5–19.0° S	142 FL [female]	--	--	--	40	Non extractive scientific fishing	[85]
Australia (GBR)	14.5–19.0° S	170 TL [female]	118 TL [male]; 130–142 TL [female]	54–61 TL	1–4	199	Commercial and scientific reef-line fisheries	[44]
Australia (northeast)	14.0° S	182 TL [female]	--	--	--	27	Non extractive scientific fishing	[86]
Australia (northern)	10.0–20.0° S	178 TL [female]	137–140 TL [female]	63 TL	2–3	94	Commercial gillnet fishery and research cruises	[87]
Australia (southern GBR)	23.5° S	150 FL [female]	--	--	--	28	Non extractive scientific fishing	[88]
Johnston Atoll	17.0° N	135 FL [male]	--	--	--	25	Non extractive scientific fishing	[89]
Johnston Atoll	17.0° N	147.4 TL [female]^b^	--	--	--	25	Cooperative shark research and control program	[40]
Madagascar	12.0° S	>170 TL [both]	--	--	--	134	Commercial gillnet fishery	[90]
Marshall Islands (Enewetak)	11.0–12.0° N	200 TL [not reported]	--	--	--	31	Non extractive scientific fishing	[41]
Marshall Islands (Enewetak)	11.0–12.0° N	152.5 TL [female]^b^	85–90 PCL [male]; 96–97 PCL [female]	--	--	76	Cooperative shark research and control program	[40]
Northern Line Islands	4.0–6.0° N	152.5 TL [male]^b^	--	--	--	79	Cooperative shark research and control program	[40]
Palau	7.0° N	158 TL [female]	--	--	--	39	Non extractive scientific fishing	[91]
Palmyra Atoll	6.0° N	175.5 TL [female]	117–121 TL [male]; 126 TL [female]	--	--	1399	Non extractive scientific fishing	This study
Papua New Guinea	2.0–12.0° S	182 TL [male]	123 TL [male]; 136 TL [female]	71–73 TL	--	133	Commercial longline fishing	[45]
USA (MHI and NWHI)	18.5–28.5° N	190 TL [female]	120–140 TL [male]; 125 TL [female]	60 TL	3–6	367	Cooperative shark research and control program	[39]
USA (NWHI)	23.0–28.5° N	168.8 TL [not reported]	--	--	--	59	Longline fishing	[62]
USA (Hawaii)	18.5–22.0° N	187 TL [female]	--	--	3–6	274	Cooperative shark research and control program	[92]
USA (Hawaii)	18.5–22.0° N	171.8 TL [male]^c^	100 PCL	105 PCL	3–6	28	Cooperative shark research and control program	[40]

^a^ Latitude rounded to the nearest 0.5°

^b^ as *Carcharhinus menisorrah*

^c^ PCL reported; regression of PCL on TL in [40]: *PCL* = 0.78*TL* − 3.022

Fishing can also affect life history traits through reductions in abundance that can lead to compensatory (density dependent) responses. For example, Cassoff et al. [25] found an increase in growth rate for an exploited northwest Atlantic porbeagle (*Lamna nasus*) population compared to its virgin growth rate. Similarly, growth rate significantly increased for the sandbar shark (*C*. *plumbeus*) and the Atlantic sharpnose shark (*Rhizoprionodon terraenovae*) following decades of exploitation [1,2]. Shark fishing is also expected to decrease maximum size [22]. However, we again did not find a consistent relationship between fishing and any life history parameters in *C*. *amblyrhynchos*. Commercial fisheries and poaching activities have reduced *C*. *amblyrhynchos* abundance in the GBR [49,50,93] and PNG *C*. *amblyrhynchos* have been harvested as a target species since the early 1980s [47]. Yet, there was no maximum length response between fished and unfished regions (Table 4), and growth at length was highest in the NWHI (Table 3; Fig 4), a region without a commercial shark fishery. Interestingly, both commercially fished GBR and PNG sharks had higher growth at length than Palmyra’s unfished shark population (Table 3; Fig 4). This suggests that a compensatory growth response is possible for this shark species, but it is not sufficient to explain observed differences in growth rates across the species’ range.

*C*. *amblyrhynchos* growth at length was dramatically different in Palmyra and the NWHI (Table 3)–the two locations that lack commercial shark fisheries—suggesting that local ecological factors may exert significant selective pressures on *C*. *amblyrhynchos* life history traits. Both the NWHI and Palmyra have high predator densities [48,51], and intra and interspecific competition are fierce. Consumptive competition across all size and age classes is expected to reduce overall resource acquisition, diminishing growth and preventing sharks from reaching the largest sizes [94,95], consistent with our findings for Palmyra, but not the NWHI (Tables 3 and 4). In Palmyra, where growth was the slowest and growth at length was lowest, *C*. *amblyrhynchos* was the most abundant shark species in forereef habitat [37], and conspecifics do not comprise a known component of their diet [39]. In contrast, in the NWHI, where growth rate was the fastest and growth at length was highest, *C*. *amblyrhynchos* were outnumbered by the larger Galapagos shark (*C*. *galapagensis*) [48], which regularly consume other elasmobranchs [96]. While extremely rare in Palmyra, tiger sharks (*Galeocerdo cuvier*) are also common in the NWHI where elasmobranchs make up 20% of their diet [97]. A strategy of rapid growth through the juvenile stage has been suggested for both the tiger shark (*G*. *cuvier*) [61] and smalltooth sawfish (*Pristis pectinata*) [98], both of which experience size-dependent mortality due to high levels of predation. Ultimately, we hypothesize that local ecological differences likely drive the variability observed in growth rates for sharks. Resource limitations due to intraspecific competition may limit growth in *C*. *amblyrhynchos* in Palmyra, while risk of predation in the NWHI may favor a different resource acquisition strategy that results in faster growth. Future life history studies should explicitly consider how the ecology of their study system exerts selection pressures on elasmobranchs.

Assuming that *C*. *amblyrhynchos* growth is affected by density dependent factors, then reduced intraspecific competition for resources caused by fishing can also lead to compensatory reductions in natural mortality [1,99]. A key feature of our study was our ability to directly estimate a survival rate for *C*. *amblyrhynchos* at an unfished location using our tag-recapture data. Given that the Palmyra shark population is likely close to carrying capacity (Bradley, unpublished data), we would expect natural mortality to be high relative to fished locations. We did indeed find some evidence of reduced natural mortality in a population of commercially fished *C*. *amblyrhyncos*, with GBR sharks reported to have lower natural mortality (*M* = 0.04–0.17 year^-1^, depending on the type of indirect estimate of *M* used [100]) than sharks in Palmyra and the NWHI. In Palmyra, survival estimated directly from tag-recapture data was 0.74 year^-1^ (95% CI 0.70–0.78 year^-1^) equating to a 0.26 year^-1^ natural mortality rate, while natural morality estimated indirectly using the Hoenig method was only slightly lower (*M* = 0.20–0.23 year^-1^; assuming that *Z* = *M* in an unfished system). There are very few estimates of natural mortality in unfished shark populations, but our estimate was similar to *M* estimated for an unexploited population of mature porbeagle sharks (*L*. *nasus*; *M* = 0.15–0.20 year^-1^, male-female, respectively [101]). Our *M* estimate for Palmyra sharks was also nearly identical to *M* estimated for NWHI sharks using the same indirect method (*M* = 0.25 year^-1^ [83]; using data from [43,62] and again assuming that *Z* = *M* in an unfished system).

A limitation of our study is the relatively lower number of captured (and recaptured) male sharks as compared to females. The sex ratio of captured *C*. *amblyrhynchos* at Palmyra was skewed towards females, which is consistent with observations from northeastern Australia [86], and Palau [91]. However, sex ratios were skewed towards males in the Main and NWHI [39] and in PNG [45]. This likely reflects a bias in sampling location and corresponding sampling method and not a real skewed sex ratio for the entire population. Sexual segregation is a common feature observed in marine species [102], including reef-associated sharks [95,103,104]. Coastal aggregations of *C*. *amblyrhynchos* tend to be dominated by female sharks [41,91,94,105,106], while male individuals have been observed dispersing farther and exhibiting lower site fidelity [85,107]. It therefore is not surprising that near-shore handline fishing was used in all cases where female biased sex ratios were observed, and offshore or deeper water longline fishing was the primary method of sampling when a male biased sex ratio was observed. When a variety of fishing methods were employed, sex ratios were not significantly different from 50:50 in both northern Australia [87] and Madagascar [90]. Previous studies have found nearly identical growth parameters in male and female *C*. *amblyrhynchos* for both female and male skewed samples [44,45]. Therefore, we are not concerned that sampling biases influenced the results we have reported here, but significant differences have been reported in male/female life history parameters for a variety of other elasmobranchs (e.g. blacknose shark, *C*. *acronotus* [3]; dusky shark, *C*. *obscurus* [60]; tope shark, *Galeorhinus galeus* [12]). Method of capture and capture location should therefore be an important consideration in future elasmobranch studies.

In this study, we revealed significant intraspecific life history variability for an elasmobranch with a large geographic range. In addition, we have shown that when we consider the multiple potential sources of this variability—latitudinal differences, ecological context, and human impacts—many of the conclusions that have emerged from previous analyses of single drivers do not hold. Most importantly, our results highlight the need for future studies to directly consider local ecology and how it may exert unique selection pressures on elasmobranch life histories through competition and predation effects, even for wide-ranging species. Large-scale effects due to fishing and latitudinal gradients may interact in ways that prevent us from understanding and predicting life history variability in elasmobranchs, ultimately misleading conservation management initiatives. As managers must accelerate their efforts to recover overexploited populations of sharks and other overfished species, it will be increasingly important to synthesize available biological information and consider how local environmental factors, ecological context, and anthropogenic impacts exert varying selection pressures on life history parameters. In the meantime, the substantial, but not yet predictable variability in life history traits observed for *C*. *amblyrhynchos* across its geographic range suggests that regional management may be necessary to set sustainable harvest targets and to recover this and other shark species globally.

## Supporting information

S1 Appendix Life history models. (PDF)

S1 Table Capture-recapture data for *Carcharhinus amblyrhynchos* caught at Palmyra Atoll used to estimate the Francis growth model. (PDF)
