# Supplementary material for: Growth and life history variability of the grey reef shark (Carcharhinus amblyrhynchos) across its range
Source: PLoS One. 2017 Feb 16;12(2):e0172370. doi: 10.1371/journal.pone.0172370 (PMC5313192; doi:10.1371/journal.pone.0172370)
Supplement: S1 Appendix — (PDF) [file pone.0172370.s001.pdf]

Supporting Information

S1 Appendix Life history models

Title: Growth and life history variability of the grey reef shark (*Carcharhinus amblyrhynchos*) across its range

Darcy Bradley, Eric Conklin, Yannis P. Papastamatiou, Douglas J. McCauley, Kydd Pollock, Bruce E. Kendall, Steven D. Gaines, Jennifer E. Caselle

1. Frisk, Miller, and Fogarty (2001) length at maturity

Frisk, Miller, and Fogarty [1] quantified the relationship between body size (total length) and length at maturity and age at maturity for 150 elasmobranch species including requiem sharks. Length at maturity L_m was significantly related to maximum length L_{max}

$$L_m = 0.70\, L_{max} + 3.29. \tag{1.1}$$

The linear relationship between L_m and L_{max} is particularly strong for individuals with $L_{max} < 200$ cm, which includes *C. amblyrhynchos*.

2. Francis (1988) growth model

The Francis [2] formulation of the von Bertalanffy growth function (VBGF) for tag-recapture data describes the expected growth from a fish of initial length *L* over some time period ΔT:

$$\Delta L = \frac{\frac{\beta g_\alpha - \alpha g_\beta}{(g_\alpha - g_\beta) - L}}{1 - \left(1 + \frac{g_\alpha - g_\beta}{\alpha - \beta}\right)^{\Delta T}}, \tag{2.1}$$

where g_α and g_β are the mean annual growth increments of a species at reference lengths α and β (which should be chosen to include a substantial proportion of by the tagging data within their range). We set ΔT=1 and standardized growth to an annual timestep. Parameters g_α and g_β can be used to estimate the conventional parameters L_∞ and k of the VBGF by the equations

$$L_\infty = \frac{\beta g_\alpha - \alpha g_\beta}{(g_\alpha - g_\beta)}; \quad (2.2)$$

$$k = -Ln\left(1 + \frac{g_\alpha - g_\beta}{\alpha - \beta}\right). \quad (2.3)$$

The Francis model is flexible in that it allows the addition of additional parameters. Assuming that the growth of a shark of length L over some time period is normally distributed with mean μ and standard deviation σ, then growth variability can be described using a single parameter *v* where

$$\sigma = v\,\mu. \quad (2.4)$$

If this mean-variance relationship results in inadequate model fit, then additional parameters can be introduced [2], but this was not necessary for our data. Outliers can also bias growth model parameters, but may represent true values that should not necessarily be discarded. The contamination probability *p* can be added to ensure that extreme data points have minimal effect on growth parameters (as long as outliers are somewhat rare). Finally, mean *m* and standard deviation *s* of measurement error in ΔL can be modeled, and the log likelihood function can be rewritten as

$$\lambda = \sum_{i=1}^{n} log[(1-p)\lambda_i + p/R], \quad (2.5)$$

where $\lambda_i = \exp\frac{-0.5(\Delta L_i - \mu_i - m)^2/(\sigma_i^2 + s^2)}{[2\pi\,(\sigma_i^2 + s^2)]^{0.5}}$ (2.6)

R is the range of observed growth increments ΔL_i and the likelihood is summed over all observed growth increments. We estimated the model using the *grotag* function with limited memory, bound-constrained BFGS maximization in the *fishmethods* package [3] to find the set of parameters that maximizes λ.

3. Jolly-Seber annual survival (ϕ)

Royle and Dorazio [4] formulated the Jolly-Seber (JS) for capture-recapture data as a restricted dynamic occupancy model where individuals can be in one of three states: "not yet entered", "alive", "dead" [5]. Transitions between these states are determined by the ecological processes entry and survival, which are estimated. We were interested in the probability of annual survival ϕ, and so we estimated a model with an annual time step where the state of an individual *i* in the first year is

$$z_{i,1} \sim Bernoulli(\gamma_1), \tag{3.1}$$

where γ is the probability that a "not yet entered" individual enters the population, and $z_{i,t} = 1$ if an individual is "alive" and present, and $z_{i,t} = 0$ if an individual is "dead" or has "not yet entered" the population [5]. Subsequent states of each individual are determined by survival for live individuals already in the population ($z_{i,t} = 1$) or by recruitment to the population for a new individual ($z_{i,t} = 0$) such that

$$z_{i,t+1} \mid z_{i,t}, \dots, z_{i,1} \sim Bernoulli\left(z_{i,t}\phi_{i,t} + \gamma_{t+1} \prod_{k=1}^{t}(1 - z_{i,k})\right), \tag{3.2}$$

where $\phi_{i,t}$ is the probability of survival for individual *i* between year *t* and *t* + 1. The observation process conditions on the above state process as

$$y_{i,t} \mid z_{i,t} \sim Bernoulli(z_{i,t} p_{i,t}), \tag{3.3}$$

where p is the probability of capture. We used a Bayesian analysis and specified uniform priors U(0,1) for all estimated parameters (ϕ, γ, p) to express our ignorance about their values [5]. The model was formulated in the JAGS language with Markov chain Monte Carlo (MCMC) sampling available in the R package *rjags* [6].

4. Hoenig (1983) total mortality (*Z*)

The Hoenig [7] method of estimating total mortality (*Z*) is parameterized around the observed relationship between longevity (T_{max}) and mortality. The equation takes the form

$$Ln(Z) = a + b\, Ln(T_{max}), \tag{4.1}$$

where *a* and *b* are fitted parameters, and T_{max} is the maximum observed age in the catch. The equation is parameterized separately for teleost fishes (a = 1.46, b = -1.01) and cetaceans (a = 0.941, b = -0.873), both of which have been used for sharks [8,9]. We assumed that *Z* was equal to natural mortality *M* given the absence of fishing at Palmyra. T_{max} was estimated as the time required to attain >99% of TL_∞ as $T_{max} = 5 \cdot Ln(2) \cdot k^{-1}$ [61], using the *k* estimate from equation 2.3.

References

1. Frisk MG, Miller TJ, Fogarty MJ. Estimation and analysis of biological parameters in elasmobranch fishes: A comparative life history study. Can J Fish Aquat Sci. 2001;58: 969–981. doi:10.1139/cjfas-58-5-969

2. Francis RICC. Maximum likelihood estimation of growth and growth variability from tagging data. New Zeal J Mar Freshw Res. 1988;22: 43–51. doi:10.1080/00288330.1988.9516276

3. Nelson GA. fishmethods: Fishery science methods and models in R. R package version 1.6-0. 2014.

4. Royle JA, Dorazio RM. Hierarchical modeling and inference in ecology: the analysis of data from populations, metapopulations and communities. Hierarchical Model Inference Ecol Anal Data from Popul Metapopulations Communities. 2008; 464. doi:10.1016/B978-0-12-374097-7.00020-X

5. Kéry M, Schaub M. Bayesian Population Analysis using WinBUGS. Bayesian Population Analysis using WinBUGS. 2012. doi:10.1016/B978-0-12-387020-9.00014-6

6. Plummer MM. rjags: Bayesian graphical models using MCMC. R Packag version 3-14. 2014.

7. Hoenig J. Empirical use of longevity data to estimate mortality-rates. Fishery Bulletin. 1983; 898–903. doi:10.2307/1940298

8. Smith SE, Au DW, Show C. Intrinsic rebound potentials of 26 species of Pacific sharks. Mar Freshw Res. 1998;49: 663-678. doi:10.1071/MF97135

9. Hisano M, Connolly SR, Robbins WD. Population growth rates of reef sharks with and without fishing on the great barrier reef: robust estimation with multiple models. PLoS One. 2011;6: e25028. doi:10.1371/journal.pone.0025028
