# Supplementary material for: Growth and life history variability of the grey reef shark (Carcharhinus amblyrhynchos) across its range
Source: PLoS One. 2017 Feb 16;12(2):e0172370. doi: 10.1371/journal.pone.0172370 (PMC5313192; doi:10.1371/journal.pone.0172370)
Supplement: S1 Table — (PDF) [file pone.0172370.s002.pdf]

Supporting Information

S1 Table

Title: Growth and life history variability of the grey reef shark (*Carcharhinus amblyrhynchos*) across its range

Darcy Bradley, Eric Conklin, Yannis P. Papastamatiou, Douglas J. McCauley, Kydd Pollock, Bruce E. Kendall, Steven D. Gaines, Jennifer E. Caselle

S1 Table. Capture-recapture data for *Carcharhinus amblyrhynchos* caught at Palmyra Atoll used to estimate the Francis growth model.

Sex	Tag Date	Recapture Date	TL-1 (cm)	TL-2 (cm)
F	07/30/07	09/27/14	103	149.5
F	07/30/07	10/02/14	130	137
F	07/30/07	11/07/08	155	154
F	07/30/07	09/26/13	155	159
F	08/01/07	11/11/08	145	152
F	08/01/07	07/09/09	145	151
F	08/01/07	11/07/08	155	158
F	08/01/07	11/07/08	156	152
F	09/07/07	07/11/09	145	146
F	09/07/07	06/02/14	145	152
F	09/07/07	05/30/13	151	156
F	09/07/07	11/07/08	164	166
F	09/07/07	03/30/11	164	171
F	07/30/08	05/29/14	134	148.5
F	11/07/08	09/27/14	155	164
F	11/07/08	05/30/13	155	161
F	11/07/08	07/11/09	156	159
F	11/07/08	07/14/09	156	155
F	11/07/08	09/27/14	156	155
F	11/07/08	07/18/13	157	161
F	11/07/08	05/30/13	160	161
F	11/07/08	09/26/13	160	159
F	11/07/08	05/29/14	160	156.5
F	11/07/08	07/11/13	160	159
F	11/07/08	05/28/13	161	162
F	11/07/08	09/26/13	166	165
F	11/11/08	10/06/14	151	163
F	11/11/08	10/02/14	155	150
F	11/11/08	10/06/14	161	158
F	11/11/08	07/09/09	167	166
F	11/12/08	09/18/13	126	135
F	11/12/08	10/06/14	126	135
F	11/12/08	05/25/13	149	150
F	11/12/08	10/06/14	154	156
F	11/12/08	07/18/13	160	158
F	11/12/08	10/06/14	166	159
F	11/14/08	07/14/09	124	127

F	11/14/08	10/06/14	124	143
F	11/14/08	09/26/13	157	163
F	11/14/08	05/30/14	157	160
F	11/15/08	07/09/09	158	158
F	11/15/08	09/20/13	163	163
F	11/15/08	05/24/14	164	164
F	07/09/09	10/02/14	130	143
F	07/09/09	09/26/13	134	137
F	07/09/09	10/02/14	137	139
F	07/09/09	09/26/13	143	149
F	07/09/09	10/06/14	143	149
F	07/09/09	09/18/13	152	161
F	07/09/09	10/02/14	152	154
F	07/11/09	10/02/14	131	140
F	07/11/09	09/14/13	136	148
F	07/11/09	05/30/14	141	152
F	07/11/09	09/27/14	144	151
F	07/11/09	05/30/13	146	150
F	07/11/09	05/29/13	165	163
F	07/11/09	09/15/15	165	163
F	07/14/09	05/30/13	152	158
F	09/13/10	05/30/13	151	151
F	09/15/10	07/16/13	133	135
F	08/03/11	07/06/13	146	147
F	08/03/11	10/01/14	146	146
F	08/03/11	09/17/13	159	157
F	08/29/12	07/05/13	157	158
F	05/25/13	09/15/15	160	161
F	05/26/13	10/03/14	163	158
F	05/27/13	05/25/14	138.5	147
F	05/27/13	09/28/14	157	160
F	05/28/13	06/05/14	144	149
F	05/28/13	09/28/14	144	145
F	05/28/13	10/02/14	154	150
F	05/28/13	10/02/14	158	156
F	05/28/13	10/07/14	160	157
F	05/29/13	10/02/14	140	138
F	05/29/13	10/02/14	144	147
F	05/30/13	06/04/14	139	143
F	05/30/13	10/06/14	148	149
F	05/30/13	10/02/14	155	159
F	05/30/13	09/15/15	155	156
F	05/30/13	09/15/15	155	157

F	05/30/13	05/29/14	162	158
F	05/30/13	10/06/14	162	162
F	05/30/13	09/15/15	162	163
F	06/06/13	10/06/14	156	160
F	06/06/13	05/24/14	162	163
F	07/06/13	10/01/14	154	154
F	07/06/13	10/01/14	162	163.5
F	07/06/13	10/01/14	168	167
F	07/18/13	06/04/14	138	139
F	09/14/13	09/30/14	140	143
F	09/16/13	09/15/15	137	141
F	09/16/13	09/30/14	154	152
F	09/18/13	09/15/15	161	160
F	09/23/13	09/29/14	157	160
F	09/25/13	09/29/14	154.5	157
F	09/26/13	10/06/14	139.5	140
F	09/26/13	10/06/14	156.5	157
F	09/26/13	05/26/14	166	164
F	05/29/14	09/15/15	134	140
F	05/31/14	09/15/15	155	157
M	10/14/06	09/22/13	100	126
M	09/11/10	10/07/14	128	134
M	09/17/10	09/24/13	147	145
M	08/03/11	09/16/13	129	132
M	05/27/13	05/25/14	147	147
M	07/09/13	05/27/14	139	136.5
M	07/09/13	10/06/14	139	141
M	07/09/13	05/27/14	143	144
M	07/16/13	10/01/14	134	145
M	09/16/13	05/26/14	143	142.5
M	09/17/13	10/01/14	159	159
M	09/20/13	06/05/14	142	148
M	09/20/13	06/03/14	144	142
M	09/20/13	06/03/14	144	143.5
M	09/20/13	05/25/14	151	148
M	09/20/13	06/03/14	157	157
M	09/25/13	10/04/14	155	156
M	09/26/13	05/31/14	105	110
